# Assessment of Median and Mean Survival Time in Cancer Clinical Trials

**DOI:** 10.1001/jamanetworkopen.2023.6498

**Published:** 2023-04-03

**Authors:** Ananya Das, Timothy A. Lin, Christine Lin, Tomer Meirson, Zachary R. McCaw, Lu Tian, Ethan B. Ludmir

**Affiliations:** 1 The University of Texas MD Anderson Cancer Center, Houston; 2 Johns Hopkins University School of Medicine, Baltimore, Maryland; 3 Azrieli Faculty of Medicine, Bar-Ilan University, Ramat Gan, Israel; 4 Insitro, South San Francisco, California; 5 Department of Health Research and Policy, Stanford University, Stanford, California

## Abstract

This cohort study assesses the relative stability of median and mean survival time estimates reported in cancer clinical trials.

## Introduction

Clinical trials often report the estimated median survival times to facilitate interpretation. However, the median makes limited use of the survival curve, focusing on the center to the exclusion of other information.^1,2^ Concerns exist about the median’s stability, as measured by the width of its CIs.^3^ In trials with few events or limited follow-up, the median survival may not be reached. In contrast, the restricted mean survival time (RMST) provides a clinically interpretable and global summary of survival and may be more stable than the median.^3,4^ The RMST represents a patient’s expected duration of survival over the follow-up period. For example, if the 5-year RMST of a treatment is 4 years, then a patient receiving that treatment would be expected to survive an average of 4 years over a 5-year follow-up period.

Instability can obscure the clinical interpretation of a survival estimate. Wide CIs suggest that the true estimate falls within a broad range of possible values, creating uncertainty and limiting the usefulness of such survival estimates for clinical decision-making. To determine the relative stability of the estimated mean and median survival times, we conducted a cohort study in which we analyzed reconstructed individual patient–level data from Kaplan-Meier curves from a comprehensive collection of 203 phase 3 cancer clinical trials.

## Methods

This study was exempt from institutional review board approval and informed consent by the Common Rule given its exclusive use of publicly available information; no patient health information was used. This study adhered to the reporting requirements of the Strengthening the Reporting of Observational Studies in Epidemiology (STROBE) reporting guideline.

All registered phase 3 cancer-specific interventional trials in ClinicalTrials.gov with reported results through 2020 were identified.^5^ Two-arm superiority trials with available Kaplan-Meier curves were selected, and individual patient–level data were reconstructed.^6^ For each trial, the median and RMST, together with the 95% CI, were estimated for each treatment arm; median and mean survival time differences, with associated CIs, were also calculated. RMST differences were calculated up to the earlier of the last events from each treatment arm. For each individual survival curve, the CI width (CIW) was calculated as the difference between the upper and lower limits of the 95% CI, and the ratio of the median and mean CIW (ie, CIW_Median_ / CIW_Mean_) was calculated. Similarly, the ratio of the CIW for the difference in median and mean survival times (ie, CIW_ΔMedian_ / CIW_ΔMean_) was calculated. The Wilcoxon signed-rank test was used to compare the CIW_ΔMedian_ and CIW_ΔMean_ across trial characteristics, with 2-tailed *P* < .05 considered statistically significant. Data were analyzed using SPSS Statistics version 28.0 (IBM) from October 4 to December 19, 2022.

## Results

Data from 203 Kaplan-Meier comparisons were eligible for analysis (Figure). The median (IQR) CIW_Median_ / CIW_Mean_ was 1.34 (1.04-1.80; *P* < .001), indicating that CIW was an average of 34% larger for median vs mean survival. Similarly the median (IQR) CIW_ΔMedian_ / CIW_ΔMean_ was 1.50 (1.15-1.99; *P* < .001).

**Figure.**
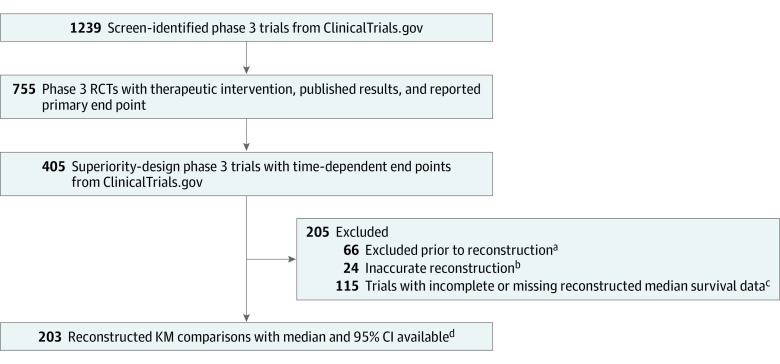
Clinical Trial Screening, Inclusion, and Results KM indicates Kaplan-Meier; RCT, randomized controlled trial. ^a^Related to missing data in the KM graph, most commonly a lack of available numbers-at-risk, which are required for reconstruction of individual patient-level data. ^b^Reconstruction accuracy was assessed by comparing the reconstructed hazard ratio value with the trial’s reported hazard ratio. If the absolute value of the difference in the natural logarithm of the reported hazard ratio and reconstructed hazard ratio was less than 0.1, then the reconstruction was considered accurate. ^c^Most trials (113) with incomplete median survival data had median survival times that could not be computed, likely as a result of one or both trial groups not reaching its median survival. Two trials were excluded due to artifacts of reconstruction that precluded the calculation of the ratio of the CIW_ΔMedian_ / CIW_ΔMean_. ^d^Three additional KM comparisons from trials with coprimary end points were included in the final tally.

We then analyzed CIW_ΔMedian_ / CIW_ΔMean_ across a spectrum of trial characteristics. The median (IQR) CIW_ΔMedian_ / CIW_ΔMean_ was 1.50 (1.13-2.15) for chemotherapy trials, 1.48 (1.12-1.96) for targeted therapy trials, and 1.36 (1.12-1.82) for immunotherapy trials. Similarly, the median (IQR) CIW_ΔMedian_ / CIW_ΔMean_ remained significantly greater than 1 across disease sites, sponsorship, primary end point selection, primary end point success, and the presence or absence of proportional hazards (Table).

**Table.** Ratio of the CIW for the Difference in Median and Mean Survival Times by Trial Characteristic

Trial characteristic	Trials, No. (%)	CIW_ΔMedian_ / CIW_ΔMean_, median (IQR)	Wilcoxon signed-rank test, *P* value
Disease site			
Breast	30 (14.8)	1.31 (1.07-1.63)	<.001
Thoracic	52 (25.6)	1.38 (1.13-1.83)	<.001
Gastrointestinal	43 (21.2)	1.41 (1.09-1.68)	<.001
Hematologic	24 (11.8)	1.82 (1.48-2.81)	<.001
Genitourinary	22 (10.8)	1.92 (1.32-2.47)	<.001
Other^a^	32 (15.8)	1.52 (1.07-2.13)	<.001
Trial therapy^b^			
Chemotherapy	35 (17.2)	1.50 (1.13-2.15)	<.001
Targeted	142 (70)	1.48 (1.12-1.96)	<.001
Immunotherapy	16 (7.9)	1.36 (1.12-1.82)	<.001
Industry sponsored			
Yes	192 (94.6)	1.50 (1.17-1.96)	<.001
No	11 (5.4)	1.38 (0.99-3.04)	.026
Cooperative group			
Yes	25 (12.3)	1.61 (1.23-2.56)	<.001
No	178 (87.7)	1.48 (1.13-1.93)	<.001
Primary end point met			
Yes	115 (56.7)	1.37 (1.13-1.77)	<.001
No	88 (43.3)	1.59 (1.17-2.1)	<.001
Primary end point^c^			
Overall survival	86 (42.4)	1.41 (1.17-1.77)	<.001
Progression-free survival	108 (53.2)	1.52 (1.11-2.15)	<.001
Proportional hazards assumption violated^d^			
Yes	50 (24.6)	1.54 (1.17-1.99)	<.001
No	153 (75.4)	1.46 (1.13-1.98)	<.001

Abbreviation: CIW, CI width.

^a^ Includes central nervous system, skin, neuroendocrine, gynecologic, sarcoma, and head and neck malignant neoplasms.

^b^ Categories with a sample size of fewer than 10 trials, (ie, 6 hormone therapy trials and 4 other therapy trials) were excluded from analysis.

^c^ Other primary end points included time to tumor progression (5 trials), time to first skeletal-related event (2 trials), bone metastases–free survival (1 trial), and survival without grade 3 to 4 toxic effects (1 trial).

^d^ The proportional hazards assumption was assessed for each trial with Schoenfeld residuals, with a *P* < .05 indicating a proportional hazards violation.

## Discussion

In this cohort study examining a comprehensive collection of phase 3 oncology trials, we observed a significant difference in the CIWs for median vs mean survival times. These data demonstrate the relative stability of the estimated mean vs median survival times across several trial characteristics, including comparisons of trials involving immunotherapy or targeted therapy. Mean and median survival are distinct summary measures of time-to-event end points, and neither alone can always adequately describe a study cohort. However, narrower CIs are desirable because they signify that a trial’s results are known more precisely. For a given treatment arm, if the CI for the RMST is narrower than that of the median, then the estimate of RMST is expected to be more precise than median survival, thereby providing greater power for detection of a treatment difference. This may have trial design implications: a trial based on the difference in median survival would need to enroll more patients to provide the same power for detecting the treatment effect than one based on the difference in RMST.

Study limitations include the necessity of using reconstructed rather than primary trial data, although only trials meeting a threshold accuracy in reconstruction compared with the reported primary results were included. Additionally, this study only examines trials from ClinicalTrials.gov, a single trial registry that may not capture all published trials. Despite these limitations, our study found a clear and consistent trend of mean survival being more stable than median survival. We encourage trialists to consider reporting the mean survival, due to its stability (narrower CIs), global character (incorporating information from across the survival curve), and interpretability.

## Notes

Supplement.
Data Sharing Statement
