# Supplementary material for: Assessment of Median and Mean Survival Time in Cancer Clinical Trials
Source: JAMA Netw Open. 2023 Apr 3;6(4):e236498. doi: 10.1001/jamanetworkopen.2023.6498 (PMC10071342; doi:10.1001/jamanetworkopen.2023.6498)
Supplement: Supplement. — Data Sharing Statement [file jamanetwopen-e236498-s001.pdf]

Data Sharing Statement

Das. Assessment of Median and Mean Survival Time in Cancer Clinical Trials. *JAMA Netw Open*. Published April 03, 2023. doi:10.1001/jamanetworkopen.2023.6498

Data
Data available: No
